# Expressive Brand Relationship, Brand Love, and Brand Loyalty for Tablet PCs: Building a Sustainable Brand

**DOI:** 10.3389/fpsyg.2020.00231

**Published:** 2020-03-06

**Authors:** Shikun Zhang, Michael Yao-Ping Peng, Yaoping Peng, Yuan Zhang, Guoying Ren, Chun-Chun Chen

**Affiliations:** ^1^College of Economics and Management, Shangqiu Normal University, Shangqiu, China; ^2^School of Business Administration, Jimei University, Xiamen, China; ^3^Business School, Yango University, Fuzhou, China; ^4^College of Economics and Management, Xi’an University of Posts & Telecommunications, Xi’an, China; ^5^Business School, Beijing Normal University, Beijing, China; ^6^School of Management, Beijing Union University, Beijing, China

**Keywords:** brand relationship, brand trust, brand loyalty, brand love, structural equating modeling

## Abstract

This study was conducted from the strategic marketing perspective to test the impact of brand relationship types on brand loyalty. We also test three path effects of brand love and brand trust. Data were collected from three metropolitan customers who use tablet PCs. We obtained 383 valid samples, giving a valid response rate of 89%. Data analysis was performed with SmartPLS2.0 and SPSS 23.0 to test the proposed model. The results indicate that an expressive brand relationship significantly predicts brand trust and brand loyalty. In turn, brand trust has a positive influence on brand love, while brand awareness and brand love influence attitudinal and behavioral loyalty. Expressive brand relationship has two indirect mediating affects via brand trust and brand love, which influence brand loyalty. Finally, we suggest managerial implications and directions for future research.

## Introduction

Computer-related products bring great changes to people’s lives, and the brand war between companies such as HP, Dell, Apple, Lenovo, ASUS, and so on is becoming increasingly intense. The computer-related product industry in Taiwan has often held a dominant market position based on superior manufacturing and branding. Several firms, including Acer and ASUS, are committed to building global brands and have recognized the importance of their own brands in the global market. Taiwan has also been a place where OEMs of international known brands gather, such as Dell, Apple, and HP (Chang et al., 2008). This implies that the relationship between customers and brands has also become an important issue for the academia and practice in terms of building sustainable brands in Taiwan. Thus, this study aims to improve our understanding of brand relationships by investigating involvement in a highly competitive context of computer-related products. Chandler and Owen (2002) emphasize that brands send out signals that provide symbolic meanings that meet customer needs, express customer wants, and interact with customers, thereby affecting customer behaviors. Aggarwal (2004) also points out that the interaction between customers and brands, which can be characterized as a relationship, can be explored only by personifying that relationship (Lombart and Louis, 2016; Charton-Vachet and Lombart, 2018). The social exchange theory states that interaction within the customer–brand relationship goes beyond the intuitive relationship of functional benefits, and regards the brand relationship as comprising exchange and communal relationships. The exchange relationship is based on reciprocity, while the communal relationship depends on emotion.

Brand relationship can create intangible added values and allows consumers to trust the brand (Park et al., 2009). For enterprises, brand relationship makes it clearly distinguished from other competing brands, forming intangible assets that are difficult to be imitated (Sreejesh and Roy, 2015; Ozturk et al., 2016). Previous studies indicate that brand relationship can increase brand loyalty (Chaudhuri and Holbrook, 2001) and brand equity (Faircloth et al., 2001), and can also affect attitude loyalty (Nyffenegger et al., 2014; Sreejesh and Roy, 2015). However, brand relationship was proven to be positively correlated with brand loyalty in some studies, while not correlated with brand loyalty in other studies (Lombart and Louis, 2016; Charton-Vachet and Lombart, 2018; Coelho et al., 2018). Is this unclear relationship between brand relationship and brand loyalty caused by certain mediating factors? This study constructs the relevant mediating factors between brand relationship and brand loyalty, trying to clarify this research gap. In extant studies, the brand relationship has been seen as a perception of relationship with continuous degree differences, taking emotion as the context and communal relationship as an expressive brand relationship. In this study, we explore the influence of the brand relationship on brand trust, brand love, and brand loyalty.

Trust can effectively reduce the uncertainties experienced by customers in the process of purchase decision making, and develop customers’ belief in the reliability, honesty, profession, and integrity of a brand, thereby affecting customers’ attitudinal loyalty and behavioral loyalty (Chaudhuri and Holbrook, 2001; Nyffenegger et al., 2014; Lombart and Louis, 2016). Brand trust is an important predictive variable of customer loyalty (Pan et al., 2012; Coelho et al., 2018). However, there is some controversy over whether brand trust directly affects brand loyalty (Chaudhuri and Holbrook, 2001; Lombart and Louis, 2016) or whether there are mediating factors at play (Aurier and Séré de Lanauze, 2012; Yasin and Shamim, 2013; Huang and Jian, 2015). This study seeks to answer this question.

Fournier (1998) indicates that brand love comprises a long-term relationship between customers and brands. Previous studies point out that the brand love consists of consumers’ affective attachment to a brand, which stimulates them to show continuous commitment or consistent behavior toward it, or a willingness to buy the brand at a premium (Batra et al., 2012; Heinrich et al., 2012; Albert and Merunka, 2013; Unal and Aydin, 2013). In addition, it is believed that brand love requires the most attention during economic downturns, and should be integrated with attitudinal and behavioral variables such as brand loyalty (Ahuvia and Ahuvia, 2006; Albert and Merunka, 2013). Moreover, although brand loyalty is difficult to understand and predict (Agustin and Singh, 2005), its generation is the most important goal of marketing (Chaudhuri and Holbrook, 2001).

Oliver (2010) divides the development of the brand loyalty into three stages: cognition, emotion, and action. Furthermore, past studies indicate that brand loyalty is influenced by customer-related factors such as satisfaction, trust, and commitment (Pan et al., 2012). However, there are few studies on whether these factors follow a specific order or occur among other antecedent motivational factors. Drawing on the relationship marketing theory (Aggarwal, 2004; MacInnis et al., 2009), the present study considers the brand relationship as the driving factor of brand trust (Chaudhuri and Holbrook, 2001), brand love (Ahuvia and Ahuvia, 2006; Heinrich et al., 2012) as occurring at the emotional level, and brand loyalty (Chaudhuri and Holbrook, 2001; Oliver, 2010) as arising at the action level. Starting from the emotional level of the brand relationship, this study discusses why there is a relationship between brand trust and brand love, and explores how this relationship can be built (Taylor et al., 2008). This study will complement extant theories related to brand loyalty.

From an academic point, this study differs from previous works because it approaches the consumer-expressive brand relationship from the angle of multi-mediating factors through dual anchors: the brand and the consumer. From a managerial point, this study deepens the knowledge of the relationship between consumer loyalty and expressive brand relationship, through the link that they may develop within process of cognition, emotion, and action. We will thus propose recommendations for managers regarding sustainable brand.

## Literature Review

### The Brand Relationship

Most studies on the brand relationship are qualitative. Social psychology divides the relationship into two categories: exchange and communal relationships (Clark and Mills, 2011). Aggarwal (2004) points out that the exchange relationship is based on reciprocity, while the communal relationship depends on emotion; the differences between these two relationships are reflected in the respective relational norms, which impact consumers’ attitudes and behaviors. The interpersonal nature of the brand relationship has been applied to the metaphor of marketing between customers and brands (Fournier, 1998; Aggarwal, 2004; Charton-Vachet and Lombart, 2018); however, brand relationship is not as easy to define and operate as is brand image. Although interpretive or qualitative methods or case studies can be applied to the subject, there is a lack of agreed-upon concepts that can be incorporated into measurable scales. The brand relationship model used here is based on Blackston’s (2000) study, which refers to the interaction between consumers’ attitudes toward brands and brands’ attitudes toward customers (Coelho et al., 2018).

Past research clearly shows that relationships are an important core of both psychology and marketing (Morgan and Hunt, 1994; Garbarino and Johnson, 1999; Delgado-Ballester and Munuera-Aleman, 2001). Chandler and Owen (2002) study the brand relationship via qualitative research, arguing that brands comprise meaning systems. This view emphasizes that the confirmation and differentiation signals sent by the brand can provide symbolic meanings that meet customer needs, express customer wants, and interact with customers, thereby affecting customer behaviors. Aggarwal (2004) also points out that the interaction between customers and brands can be explored only by personifying the brand relationship. Based on the law of reciprocity, Fournier (1998) suggests that brand relationship quality comprises six aspects, which can be used as a reference for strengthening the brand relationship. These aspects are love and passion, self-connection, commitment, interdependence, intimacy, and brand partner quality. In addition, Fournier (1998) outlines 15 categories of brand relationships, which can, in turn, be depicted as expressive or instrumental brand relationships—though the two types are not mutually exclusive. Exchange relationships are economic in nature and provide utilitarian benefits, while communal relationships entail emotions toward others that transcend self-interest. Trust arises as a result and forms the cornerstone of close relationships within psychology and marketing (Garbarino and Johnson, 1999). Esch et al. (2006) argue that the brand relationship is composed of brand satisfaction, brand trust, and brand attachment, but such a simulation is a concept concerning the quality. Scholars also regard dependence and behavioral loyalty as part of the brand relationship (Fournier, 1998; Oliver, 1999; Davis et al., 2009).

### Brand Trust

Doney and Cannon (1997) emphasize that brand trust is the degree to which customers believe that a brand can provide the required value (Chaudhuri and Holbrook, 2001; Charton-Vachet and Lombart, 2018; Coelho et al., 2018). Doney and Cannon (1997) believe that brand trust plays an important role in long-term customer relationships and that brand trust can reduce the uncertainty customers feel about a product when finding it difficult to make a purchase decision (Charton-Vachet and Lombart, 2018). Chaudhuri and Holbrook (2001) define brand trust as the customer’s belief that a brand has the ability to perform its claimed functions. Chen and Hu (2010) also point out that trust is an expectation or belief—i.e., customers’ belief that services purchased will provide reliable and as-promised performance. Delgado-Ballester and Munuera-Aleman (2001) classify brand trust into reliability and intention, arguing that brand trust comprises an awareness of the brand’s trustworthiness and an expectation that the brand will fulfill its obligations and responsibilities. They also point out that the brand is not only a product but also an important partner in the relationship between customers and brands. On this basis, this study defines brand trust as the customer’s awareness of the brand’s kindness and integrity (Aurier and N’Goala, 2010; Dwivedi and Johnson, 2013; Coelho et al., 2018).

Blackston (2000) points out that interaction takes place between consumers’ attitudes toward brands and brands’ attitudes toward consumers, and finds that successful brand relationships entail trust and satisfaction (Nyffenegger et al., 2014; Charton-Vachet and Lombart, 2018). The exchange relationship is based on reciprocity, while the communal relationship depends on emotion; the differences between these two relationships are reflected in the respective relational norms, which impact consumers’ attitudes and behaviors (Aggarwal, 2004; Coelho et al., 2018). In the business context, the communal relationship involves people’s emotions that go beyond self-interest, while an expressive brand relationship is based on the contact referred to in the social exchange theory; the benign interaction within an expressive brand relationship can determine consumers’ trust in the brand. In this regard, the following hypothesis is posited:

H1: An expressive brand relationship has a positive impact on brand trust.

### Brand Love

Ahuvia and Ahuvia (2006) believe that when a brand maintains and develops a sustainable trading relationship with its customers, knowing whether it can satisfy the emotional needs of customers will help it to predict or explain customer behavior and generate high satisfaction. On the basis of the triangular theory of interpersonal love (Sternberg, 1997), and referencing a study by Heinrich et al. (2012), we use “brand commitment,” “brand closeness,” and “brand enthusiasm” as variables to measure brand love. We suggest that the relationship between the customer and a brand will change from satisfaction to love when a customer connects to the brand and considers it a manifestation of their self-identification (Ahuvia and Ahuvia, 2006; Unal and Aydin, 2013). Since the customer believes the brand to be reliable and trusts in the promises the brand makes (Sirdeshmukh et al., 2002), brand trust can reduce uncertainty related to customers’ purchases (Gommans et al., 2001) and strengthen the emotional antecedents (Heinrich et al., 2012). Chaudhuri and Holbrook (2001) point out that brand trust and brand affect are important factors impacting brand loyalty, though the specific relationship is not clearly indicated. Song et al. (2012) highlight that brand affect influences brand trust. Brand trust positively influences brand enthusiasm (Albert and Merunka, 2013), which is one of the components of brand love, and brand trust positively impacts brand love (Albert and Merunka, 2013; Huang and Jian, 2015). Thus, we suggest the following hypothesis:

H2: Brand trust has a positive influence on brand love.

### Brand Loyalty

Customer purchasing behavior is not a random response, but the result of a long-term influence of customers’ inner factors. In addition to repeat purchase behaviors, customers will be committed to a brand at the psychological level. This means that in a competitive market such as that for tablet PCs, brand loyalty not only attracts new customers but also maintains ongoing purchases. In terms of measuring brand loyalty, most empirical studies state that this construct should be considered in terms of both attitude and behavior—i.e., attitudinal loyalty and behavioral loyalty (Baldinger and Rubinson, 1996; Mukherjee and Nath, 2007; Sondoh et al., 2007; Chen and Hu, 2010; Deng et al., 2010; Alireza et al., 2011; Chen et al., 2014; Charton-Vachet and Lombart, 2018). Attitudinal loyalty is the consumer’s response at the psychological level, where the customer is willing to purchase and recommend the brand’s products and services to relatives, friends, or others even if the price is higher. Behavioral loyalty is the customer’s degree of preference for the branded product or service—that is, their willingness to purchase the brand’s products or services in the future.

Sarkar and Sreejesh (2014) point out that brand love does not directly affect purchase intention, but occurs through brand jealousy. On the contrary, Ahuvia and Ahuvia (2006) suggest that brand love positively affects brand loyalty and word of mouth; the more intense the brand love, the higher the customer’s willingness to purchase products at a price premium (Chaudhuri and Holbrook, 2001; Thomson et al., 2005; Heinrich et al., 2012). In addition, brand love affects brand loyalty (Ahuvia, 2005; Ahuvia and Ahuvia, 2006; Bergkvist and Bech-Larsen, 2010; Batra et al., 2012; Chen and Quester, 2015). Thus, we posit that:

H3a: Brand love has a positive influence on attitudinal loyalty.

H3b: Brand love has a positive influence on behavioral loyalty.

The expressive brand relationship is based on the contact that occurs between customer and brand, as indicated in social exchange theory. Such benign interaction can determine the affection of customers toward brands and improve those brands’ identity (Lombart and Louis, 2016). A more intimate, continuous, and stable relationship can be formed through a personified expressive brand relationship on the basis of interaction (Aggarwal, 2004; Coelho et al., 2018). Consumers buy products due to their love for the brand. Thus, customers who have an expressive brand relationship more easily form brand loyalty, which can be classified into attitudinal and behavioral loyalty (Charton-Vachet and Lombart, 2018). Thus, we propose the following:

H4a: An expressive brand relationship has a positive influence on attitudinal loyalty.

H4b: An expressive brand relationship has a positive influence on behavioral loyalty.

**FIGURE 1:**
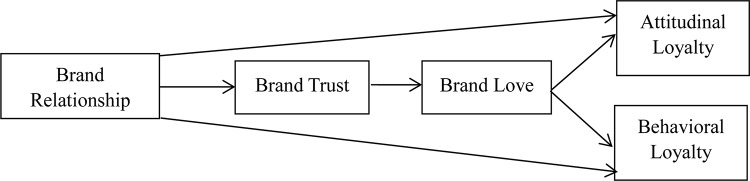
Research framework.

Drawing on relationship marketing theory (Aggarwal, 2004; MacInnis et al., 2009), we consider the expressive brand relationship as the driving factor of the brand trust (Chaudhuri and Holbrook, 2001) and brand love (Ahuvia and Ahuvia, 2006; Heinrich et al., 2012), at the emotional level, and of brand loyalty (Chaudhuri and Holbrook, 2001; Oliver, 2010) at the action level. Taking users of tablet PCs in Taiwan as our focus, a theoretical model is established to explore why the three-path mediated effect between brand trust and brand love exists, and how such an effect can be built (Taylor, 2008). The conceptual framework and the research hypothesis of this study are shown in Figure 1.

## Methodology

### Sampling

We conducted a survey using purposive sampling. This sampling method can be implemented based on the respondents’ subjective judgment to select a sample that is most suitable for the purpose of the research. The tablet PC brands selected in the survey were launched by six well-known brands in Taiwan, such as Apple, Samsung, ASUE, Acer, Lenovo, and Sony. In addition, to accurately measure consumers’ perceptions of the variables of the study, two principles for sampling were set. First, the consumers filling out the questionnaire must be users of tablet PCs to ensure the reliability of the subjects in filling out the variable items. Second, we sent the questionnaires to the brand specialty stores of tablet PCs and asked the sales clerk to give questionnaires to consumers who have already purchased tablet PCs, in order to avoid the questionnaires being filled out by consumers who have not purchased tablet PCs. The sampling time was 2 months. A total of 430 questionnaires were distributed, of which 397 were returned. After removing invalid questionnaires, wherein more than 5% of questions were unanswered and that with regular answers, a total of 383 valid questionnaires remained, giving a response rate of 89%. Tests for non-response bias were based on the comparison of early (first month) and late (second month) respondents in terms of the mean values of variables items (Armstrong and Overton, 1977). These tests yielded no significant differences, suggesting that non-response bias may not be a major problem in this study.

The respondents were mostly female (52.2%), with the majority aged between 21 and 29 (39.7%). Most users had graduated from university (59%), and their average monthly income was NTD 25,001–35,000 (26.6%). Most of the respondents’ tablet PCs were made by Apple (42.8%), and the majority lived in the north of Taiwan, accounting for 44.1%, which is consistent with the current distribution. The majority had used tablet PCs for 2 years (37.3%); they were clear to the service condition of tablet PCs, and it was not the first time they had used them. The sample was thus deemed suitable for this research. The information about the sample profile is shown in Table 1.

**TABLE 1:** Descriptive statistics.

Variable	Items	*N*	Variable	Items	*N*
Gender	Male	183	Education	High school	41
	Female	200		College	47
Age	Below 20	14		University	226
	21–29	152		Master	69
	30–39	117	Income	Below 25000	80
	Above 40	100		25001–35000	102
Brand	Apple	164		35001–45000	78
	Samsung	66		45001–55000	64
	ASUE	70		Above 55001	59
	Acer	37	Use year	Below 1 year	78
	Lenovo	28		2 year	143
	Sony	18		3 year	62
				Above 4 year	100

### Measure

The measures used in this study were as follows. Brand love was measured from the three perspectives of brand commitment, brand closeness, and brand enthusiasm, based on the study by Heinrich et al. (2012), through 12 items. Brand trust was measure using five items according to research by Chaudhuri and Holbrook (2001) and Dwivedi and Johnson (2013). Brand loyalty was measured using eight items through the two perspectives of attitudinal loyalty and behavioral loyalty on the basis of the study by Chaudhuri and Holbrook (2001). Referring to a study by Aggarwal (2004), expressive brand relationship was measured using five items, as was instrumental brand relationship. In addition, previous studies show that income affects brand love (Vlachos and Vrechopoulos, 2012), years of use affect brand loyalty (Chaudhuri and Holbrook, 2001), and consumer psychological factors such as brand awareness have a positive influence on brand loyalty (Giovannini et al., 2015). Therefore, we applied monthly income, years of use, and brand awareness as control variables.

## Results

### Assessment of the Measurement Model

To gauge the reliability and validity of the scale, we adopted confirmatory factor analysis (via AMOS) to verify both the convergent and discriminant validity. Hair et al. (2010) designated the standards of convergent validity criteria as follows: standardized factor loading higher than 0.5, average variance extracted (AVE) higher than 0.5, and composite reliability (CR) higher than 0.7. The evaluation standard for discriminant validity is the square root of the AVE for one dimension being greater than its correlation coefficient with any other dimension(s). As Tables 2, 3 show, all items in the measures of exogenous variables were significantly explained, suggesting that the items were converged to this factor, and, hence, to their corresponding dimensions. Therefore, the scale had convergent validity. Finally, as also shown in Table 2, the correlation coefficients of the dimensions were all less than the square root of the AVE, suggesting that each dimension in this study had good discriminant validity. For mode-matching tests, the ratio of χ^2^ to its df (2.47) was less than 3, and PNFI (0.7) was greater than 0.5. Goodness of fit index was 0.97, adjusted goodness of fit index was 0.94, normed fit index was 0.98, comparative fit index was 0.99, and incremental fit index was 0.99. All of them were greater than 0.9. In addition, the root mean square error of approximation was 0.05, which is less than 0.06.

**TABLE 2:** Factor loadings of measure items.

Measure variables	Items	Factor loadings
Instrumental	I choose this brand because it is not expensive	0.913
relationship	I choose this brand because of its easy promotion and discounts	0.869
Expressive relationship	I choose this brand because I like it from the heart	0.885
	I choose this brand because it is amiable	0.851
	I choose this brand because it brings me a sense of safety	0.933
	I choose this brand because I identify with its concept	0.914
	I choose this brand because its functions is satisfied with me	0.883
Brand love	Brand commitment	0.907
	Brand closeness	0.876
	Brand enthusiasm	0.841
Brand trust	I trust this brand	0.895
	I rely on this brand	0.855
	This is an honest brand	0.908
	This brand is safe	0.902
	This brand can address my concerns	0.875
Attitudinal	I am committed to this brand	0.892
loyalty	I would be willing to pay a higher price for this brand over other brand	0.922
	I identify with this brand very much	0.920
	If this brand is out of stock, I will wait and refuse any substitutes	0.903
Behavioral	I will buy this brand the next time	0.909
loyalty	I intend to keep purchasing this brand	0.911
	I will recommend the brand to others	0.830
	Overall, I buy this brand most often	0.915

**TABLE 3:** Measurement properties.

	1	2	3	4	5	6
(1) Attitudinal loyalty	0.902					
(2) Behavioral loyalty	0.756	0.890				
(3) Brand trust	0.763	0.773	0.876			
(4) Brand love	0.710	0.782	0.728	0.868		
(5) Instrumental relationship	−0.210	−0.121	−0.104	−0.093	0.852	
(6) Expressive relationship	0.723	0.763	0.732	0.724	−0.116	0.901
AVE	0.813	0.800	0.767	0.754	0.725	0.811
CR	0.945	0.932	0.950	0.893	0.879	0.944
α	0.924	0.916	0.933	0.854	0.753	0.937
Mean	4.612	3.782	3.889	3.562	2.983	3.698
*SD*	0.832	0.776	0.683	0.800	0.835	0.672

### Structural Model Results

The hypotheses were tested using partial least squares structural equation modeling (PLS-SEM). The primary advantages of PLS-SEM include the relaxation of normal distributional assumptions required by PLS-SEM’s ability to easily estimate much more complex models with smaller sample sizes (Shiau and Chau, 2016; Hair et al., 2019; Khan et al., 2019; Shiau et al., 2019). PLS-SEM is more suitable for this study under the following situations: when the research objective is exploratory research for theory development, when the analysis is for a prediction perspective; when the structural model is complex, when the structural model includes one or more formative constructs; when distribution is lack of normality, and when research requires latent variable scores for consequent analyses (Shiau and Chau, 2016; Hair et al., 2019; Khan et al., 2019; Shiau et al., 2019). The above reasons provide supports to consider the PLS as an appropriate SEM method for a study.

**FIGURE 2:**
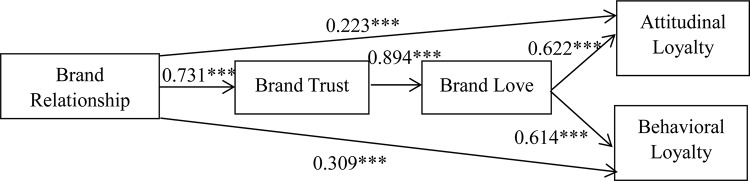
Results of SEM analysis.

This analysis showed that the proportion of variance shared exclusively with each additional variable. Figure 2 shows the results of the model’s main effect, which indicated that brand relationship (H1, H4a, H4b) has a significant positive influence on brand trust, attitudinal loyalty, and behavioral loyalty; brand trust (H2) has a significant positive influence on brand love; and brand love (H3a, H3b) has a significant positive influence on attitudinal loyalty and behavioral loyalty. The results indicate that a significant increase in the brand relationship increases brand trust (β = 0.731, *p* < 0.001), which supports H1. Likewise, brand trust significantly improves brand love (β = 0.894, *p* < 0.001), which fully supports H2. Coefficients of the correlation between brand love and attitudinal loyalty and between brand love and behavioral loyalty were 0.622 (*p* < 0.001) and 0.614 (*p* < 0.001), respectively. These positive relationships support H3a and H3b. Finally, the brand relationship was found to influence the development of attitudinal loyalty (β = 0.223, *p* < 0.001) and behavioral loyalty (β = 0.309, *p* < 0.001), supporting H4a and H4b.

### Examination of Mediating Effects

The normalized effect values of the direct, indirect, and total effects of the constructs were collated, as shown in Table 4, and path verification regarding the meditating effect was performed. The path coefficients of the indirect effect on attitudinal and behavioral through brand love and brand trust were 0.407 and 0.401, respectively. Based on suggestions by Shrout and Bolger (2002), the ratio of the indirect effect and the total effect was used as the evaluation index of indirect effect intensity; this showed that the intensity of the indirect effects were much greater than that of the direct effects (0.223 and 0.309). This indicates that the indirect effect plays an important role, and also confirms that brand love and brand trust have total mediating effects on the relationship between brand relationship and brand loyalty.

**TABLE 4:** Path coefficient of direct, indirect, and total effects.

Construct	Effects	Brand	Attitudinal	Behavioral
		love	loyalty	loyalty
Brand	Direct effect	0.731	0.223	0.309
relationship	Indirect effect	–	0.407	0.401
	Total effect	0.731	0.630	0.710
Brand trust	Direct effect	0.894	–	–
	Indirect effect	–	0.556	0.549
	Total effect	0.894	0.556	0.549
Brand love	Direct effect	–	0.622	0.614
	Indirect effect	–	–	–
	Total effect	–	0.622	0.614

## Conclusion

### Discussion

Customers are willing to purchase products at a premium price when they are loyal to the brand (Albert and Merunka, 2013), which indicates a level of brand trust. The expressive brand relationship is based on a good relationship between customer and brand, wherein a close, continuous, and stable relationship is formed on the basis of interaction (Aggarwal, 2004). This study aimed to verify whether brand relationship will positively affect brand loyalty, assuming that brand relationship has a direct effect on brand trust, attitudinal loyalty, and behavioral loyalty. The results support this assumption. With this in mind, brand managers should make every effort to build an expressive brand relationship via benign interactions with customers and create scenarios that highlight the accessibility of the brand.

In addition, the results reveal positive and direct impacts of brand love on the development of attitudinal and behavioral loyalty. Brand loyalty is formed not through the expressive brand relationship, but rather via the brand love. The main duty of brand managers is to build a contribution to their company by means of creating brand value. Thus, brand managers should make good use of the fact that expressive brand relationships have a major impact on both brand trust and brand loyalty, and actively plan and utilize various marketing strategies to ensure a closer relationship between customers and brands.

This study also discussed the mediating mechanism between brand relationship and brand loyalty. Results show that consumers’ higher awareness of brand relationship are easier to lead to brand trust and brand love with a high intensity, and this intensified brand trust and brand love will contribute to attitudinal and behavioral loyalty. Customers are willing to be loyal to a brand, and buy its products at a premium price (Albert and Merunka, 2013), if they develop trust in it, which means they form preferences for the brand and repeatedly purchase its products. Thus, brand trust and brand love can make valuable contributions (Chaudhuri and Holbrook, 2001). This conclusion is consistent with the findings of Oliver (2010) that brand trust and brand love play an important role in the cognition–emotion–action model, and have an influence on the development of brand loyalty that cannot be ignored.

Based on the above research results and discussions, this study confirms several contributions. First, this study applied the research results of relationship orientation in the Eastern social culture, serves as the theoretical basis that connects brand relationship and brand loyalty, and theoretically contributes to the construction of brand value chain. Second, this study also provides insights into how to foster a long-term behavior and attitude for brand loyalty through the brand relationship with the service-dominant logic. Third, this study discusses that the brand relationship will finally affect the brand loyalty via the mediating effect of brand trust and brand love, and verifies the importance of mediators between brand relationship and brand loyalty (Taylor et al., 2008). Fourth, brand loyalty, as the complex of behavior and attitude, indicates customer’s long-term commitment and emotional preference to specific brands, reflects the customer’s behavioral outcome, and proves the importance of marketing strategies, in addition to reflecting actual purchasing behaviors of customers.

### Theoretical Implications

Customers’ positive perceptions of a brand allow them to establish long-term relationships with that brand. This relationship enhances brand trust, brand love, and brand loyalty. Although Payne et al. (2009) present a brand relationship experience model via a case study, there is no empirical evidence of service-led logic to date. This study finds that the different forms of brand relationship have varying effects on brand loyalty and brand trust, which also indicates the importance of brand management under the service-led logic. This shows that in order to allow consumers to establish a lasting emotional relationship with a brand, brand trust and brand love models should also be incorporated to enhance brand loyalty, in addition to allowing consumers to have a psychologically intimate relationship with the brand. In general, our consideration of the brand relationship in light of service-led logic helps to guide firms on how to foster longer-term behaviors and attitudes, as well as providing empirical support for the theoretical model posited by Payne et al. (2009).

Previous studies discuss the role of brand satisfaction and brand love (Correia Loureiro and Kaufmann, 2012), but rarely verify the mediating role of brand trust and brand love in a serial way. This study confirms that there are other mediating variables affecting brand loyalty and highlights brand love as one such mediator (Aurier and Séré de Lanauze, 2012; Yasin and Shamim, 2013; Taylor et al., 2014; Huang and Jian, 2015). Brand trust and brand love are important bridges for the emotional brand relationship and brand loyalty. These important findings can be used to extend existing brand theory and echo the recommendations of Ahuvia and Ahuvia (2006) and Heinrich et al. (2012). Incorporating brand trust and brand love into the overall research model can improve predictions of brand loyalty and overcome inconsistent findings about brand trust.

Most past research applies interpersonal theories derived from a Western context in terms of a theoretical framework of the brand relationship. However, interpersonal theories that apply to China differ from those of Western countries. Brands, as tools for building relationships, should be adapted to the social environment. It has been found that Chinese tend to be affected by established relationships, while Western studies have focused on interactions between brands. On this basis, our study builds a brand relationship model based on indigenized thinking, and highlights the need to focus on the brand relationship and brand loyalty, which are of great importance. The results of this study are not only more explanatory but also provide effective guidance for solving difficulty, a difficult aspect (brand loyalty) of relationship marketing—i.e., how to build brand loyalty.

### Managerial Implications

Customers’ brand love can enhance their adaptability and positive consciousness toward the brand, thereby improving their satisfaction. To retain existing customers and improve the repurchase rate, companies not only need to maintain a reliable and expressive relationship with customers but also improve customers’ brand loyalty. The latter is key to enhancing market share. If brand operators are able to induce customers to generate passion for the brand, these customers will become brand followers who actively recommend the brand to others, creating public praise for the brand. More brand lovers means more brand loyalists, which will give the brand significant competitive advantage.

Brands should create appropriate loyalty plans to build long-term relationships with customers. Based on inherent and extrinsic incentive motivations, as well as integrated marketing communications such as via the establishment of brand communities, brands can be exposed to customers through frequent postings on social media such as Facebook. Although HTC once employed Robert Downey Jr. as its spokesperson, it was unable to deepen the topic and form a close relationship with customers, let alone promote brand trust and love, because it failed to integrate traditional channels with its online network, leading to the brand’s estrangement from customers. This case indicates that brands will be unable to establish stable expressive brand relationships with customers and further generate brand trust and love, let alone improve brand loyalty, if they ignore the core brand core values, let alone the improvement of brand loyalty.

Brand managers can choose their positioning and strategies based on their individual environmental conditions. To establish an instrumental brand relationship, brand managers can highlight product differentiation through a low-cost strategy; however, this only works in the short term, and as this study shows, the strategy will negatively affect brand trust. Thus, it must be prudent to apply this strategy. To establish an emotional brand relationship, brand managers should be good at utilizing integrated marketing communications, enrich brand connotations, conduct effective internal marketing, improve employees’ service attitude, enhance brand loyalty based on service-led logic, and develop continuous competitive advantages. Brand managers can adopt different brand strategies based on their own situations by referencing this study in order to enhance their brand position and competitive advantage.

### Limitations and Future Research

Since we used a structured questionnaire to collect our data, this study is considered cross-sectional, so no long-term data was collected regarding customers’ loyalty to tablet brands. In view of this, researchers are encouraged to explore situations in which consumers respond to the different brand relationships, and take account of topics related to brand love, brand trust, and brand loyalty from qualitative and quantitative viewpoints, to the extent permitted by data resources.

We discussed the brand relationship based on social exchange theory. Future studies should be conducted from the perspective of the importance of the brand community for the brand relationship, as per Muniz and O’guinn (2001), which will deepen findings on the application of brand management under the service-led logic. Vargo and Lusch’s (2004) service-led logic goes against the idea that firms only focus on the exchange value created by customer value. Due to continuous changes in the economic environment, economic activities and business modes are no longer just tangible and static commodities. As a result, the focus of firms is shifting from tangible assets to interactional, connected, and constant relationships, which is also consistent with this study’s emphasis on transaction process instead of transaction affair (Vargo and Lusch, 2004).

Several aspects were not measured in this study, such as brand community (Muniz and O’guinn, 2001; Baldus et al., 2015). Furthermore, social media or community engagement (Habibi et al., 2014), and social identity theory (Bhattacharya and Sen, 2003), can be considered in future research. The brand network relationship of communities relates to the interaction in, and establishment of, brand relationships, and subsequent research can analyze the brand relationship from the perspective of such social networks.

## Data Availability Statement

The raw data supporting the conclusions of this article will be made available by the authors, without undue reservation, to any qualified researcher.

## Ethics Statement

The studies involving human participants were reviewed and approved by Institutional Review Board, University of Taipei. The patients/participants provided their written informed consent to participate in this study.

## Author Contributions

This study is a joint work of SZ, MP, YP, and YZ. SZ and MP contributed to the ideas of brand relationship, collection of data, and empirical analysis. MP and YP contributed to the data analysis, design of research methods, and tables. YZ, GR, and C-CC participated in developing a research design, writing, and interpreting the analysis. All authors contributed to the literature review and conclusions.

## Conflict of Interest

The authors declare that the research was conducted in the absence of any commercial or financial relationships that could be construed as a potential conflict of interest.
