# Corrigendum: Expressive Brand Relationship, Brand Love, and Brand Loyalty for Tablet PCs: Building a Sustainable Brand

**DOI:** 10.3389/fpsyg.2020.00763

**Published:** 2020-04-23

**Authors:** Shikun Zhang, Michael Yao-Ping Peng, Yaoping Peng, Yuan Zhang, Guoying Ren, Chun-Chun Chen

**Affiliations:** ^1^College of Economics and Management, Shangqiu Normal University, Shangqiu, China; ^2^School of Business Administration, Jimei University, Xiamen, China; ^3^Business School, Yango University, Fuzhou, China; ^4^College of Economics and Management, Xi'an University of Posts & Telecommunications, Xi'an, China; ^5^Business School, Beijing Normal University, Beijing, China; ^6^School of Management, Beijing Union University, Beijing, China

**Keywords:** brand relationship, brand trust, brand loyalty, brand love, structural equating modeling

In the published article, there were several errors regarding the affiliations of authors. Instead of affiliation 1, Michael Yao-Ping Peng should have the following affiliation, which has now been added as affiliation 2:

“School of Business Administration, Jimei University, Xiamen, China”

Yaoping Peng should have affiliation 3 instead of 2. Instead of affiliation 3, Yuang Zhang should have the following affiliation, which has been added as affiliation 4:

“College of Economics and Management, Xi'an University of Posts & Telecommunications, Xi'an, China”

The authors apologize for these errors and state that these do not change the scientific conclusions of the article in any way. The original article has been updated.

